# Abundance and short-term temporal variability of fecal microbiota in healthy dogs

**DOI:** 10.1002/mbo3.36

**Published:** 2012-09-03

**Authors:** Jose F Garcia-Mazcorro, Scot E Dowd, Jeffrey Poulsen, Jörg M Steiner, Jan S Suchodolski

**Affiliations:** 1 Gastrointestinal Laboratory, Department of Small Animal Clinical Sciences, College of Veterinary Medicine, Texas A&M UniversityCollege Station, TX; 2 Molecular Research LP (MR DNA)Shallowater, TX

**Keywords:** Fecal microbiota, FISH, 454-pyrosequencing, variability

## Abstract

Temporal variations of intestinal microorganisms have been investigated in humans, but limited information is available for other animal species. The aim of the study was to evaluate the abundance and short-term temporal variability of fecal microbiota in dogs. Two fecal samples were collected (15 days apart) from six healthy dogs. The microbiota was evaluated using fluorescence *in situ* hybridization (FISH) and 454-pyrosequencing targeting the 16S rRNA and its gene. Pyrosequencing revealed 15 families comprising >80% of all microbiota, over time intraindividual coefficients of variation (%CV) ranged from 2% to 141% (median: 55%). In contrast, the interindividual %CV ranged from 62% to 230% (median: 145%). Relative proportions of *Faecalibacterium* (important for intestinal health) and *Subdoligranulum* were low (two dogs harbored 4–7% of *Subdoligranulum*, the remaining dogs had <1% of either genus). Conversely, FISH revealed that *Faecalibacterium* comprised a median of 5% of total counts (range: 0–8%, probe Fprau645). A novel FISH probe (Faecali 698) was tested that, compared with Fprau645, can detect *in silico* a similar percentage of *Faecalibacterium* but higher proportions of *Subdoligranulum*. This probe revealed a high percentage of *Faecalibacterium–Subdoligranulum* (median: 16% of total counts). Future studies should consider the observed variability and discrepancies in microbial abundance between FISH and 454-pyrosequencing.

## Introduction

The gastrointestinal (GI) tract of animals contains trillions of microorganisms that help maintain intestinal and overall health. Fueled by recent advances in the field of molecular tools and bioinformatics, the last decade witnessed an improved understanding on the phylogenetic structure of the intestinal microbiota and its symbiotic association with the host (Suchodolski 2011; Walter and Ley 2011).

The manipulation of intestinal microorganisms to improve health has been of interest for decades. However, the exact abundance and/or proportion of each member of the intestinal microbiota are a matter of debate, although some trends have been recognized for some bacterial groups (Hoyles and McCartney 2009). Also, only few studies have evaluated temporal variations of the intestinal microbiota (Franks et al. 1998; Buddington 2003; Matsuki et al. 2004; Dethlefsen and Relman 2011) and most of these studies have investigated the human microbiota only, with limited available information about the abundance and variability of the intestinal microbiota in other animal species. A recent study evaluated interindividual differences in fecal microbiota of healthy cats and dogs using 454-pyrosequencing, but only one time point was evaluated and no truly quantitative assays were performed (Handl et al. 2011). Currently available microbial identification techniques can yield distinctive views of the membership composition of the intestinal microbiota, based on their differences in estimating the abundance of the microorganisms. For example, sequencing technologies, especially high-throughput techniques, such as microarray-based methods or 454-pyrosequencing, are able to characterize in depth the composition of the intestinal microbiota but are only semi-quantitative in nature. Other techniques, such as fluorescence *in situ* hybridization (FISH), can provide the actual numbers of the microorganisms but its application to all microbiota is technically challenging. The aim of this study was to evaluate the abundance and short-term temporal variability of fecal microbiota in a population of healthy dogs using a semi-quantitative (454-pyrosequencing) and a quantitative (FISH) approach.

## Materials and Methods

### Animals and fecal collection

Six privately owned pet dogs were enrolled (Table 1). All dogs were considered healthy on physical examination by a veterinarian, lived in different households, did not have any clinical signs of GI disease nor have consumed antibiotics for at least 3 months before fecal sample collection. Serum concentrations of cobalamin, folate, pancreatic lipase immunoreactivity, and trypsin-like immunoreactivity were measured to rule out subclinical GI disease (results not shown). Naturally passed fecal samples were collected from all dogs at two time points (15 days apart) within the same time frame (Fall 2010). The study protocol was approved by the Clinical Research Review Committee (CRRC#10-14) at Texas A&M University and written informed consent was obtained from the owners of all enrolled animals.

**Table 1:** Signalment of enrolled dogs

Dog	Breed	Age	Weight (kg)	Diet
D1	Labrador mix	10 years	33	Beneful
D2	Boston Terrier	4 years	11	Science Diet Sensitive Stomach
D3	Labrador mix	1.5 years	25	Purina One
D4	Labrador mix	5 years	23	Dry food (type not specified)
D5	Mixed	4 years	23	Purina Sensitive Skin and Stomach
D6	Australian Kelpie	9 months	16	Purina High Performance

### Assessment of fecal microbiota

#### Extraction of DNA

Genomic DNA was extracted and purified from 100 mg of each fecal sample using a phenol–chloroform extraction procedure as described by Suchodolski et al. (2005).

#### 454-Pyrosequencing

The fecal bacterial taxonomic structure was evaluated using a bacterial tag-encoded FLX-titanium 16S rRNA gene amplicon pyrosequencing (bTEFAP) as described previously for canine fecal samples (Handl et al. 2011). The sequence processing pipeline was carried out as described in detail by Garcia-Mazcorro et al. (2012). Briefly, Phred20 quality reads were trimmed to remove tags and primer sequences, depleted of non-16S rRNA reads, chimeric, mitochondrial, and sequences of less than 250-bp length. The final sequences were evaluated using a standard nucleotide basic local alignment search tool (BLASTN) against a continually curated high-quality 16S rRNA gene database derived from the National Center for Biotechnology Information (NCBI). BLAST outputs were compiled to generate percentage files at each taxonomic level. Sequences with identity scores to known of well-characterized 16S rRNA gene sequences between 95% and 97% identity were resolved at the genus level, between 90% and 95% at the family level, between 85% and 90% at the order level, between 80% and 85% at the class level, and 77–80% at the phylum level. Sequence information is available through GenBank within a shortread archive (SRA) under accession (SRA053134).

#### Fish

For FISH analysis, we obtained an aliquot of 100 mg from each fecal sample and prepared paraffin-embedded fecal blocks (PEFB). These blocks were prepared because currently available FISH protocols often do not allow for a consistent and uniform distribution of the microorganisms across the glass slide and are associated with a low throughput. A detailed description of the procedure to prepare the fecal blocks from 100 mg of feces (wet weight) is provided as supplementary information.

For the FISH procedure, two serial sections (5 *μ*m) of each fecal block were cut and placed on coated glass slides (IMEB Inc., San Marcos, California). The sections were deparaffinized by passage through xylene (3 × 10 min), 100% ethanol (2 × 5 min), 95% ethanol (1 × 5 min), and 75% ethanol (1 × 5 min). After the slides were air-dried, a total of 10 *μ*L of warm hybridization buffer (containing 30 ng/*μ*L of the respective probe, each labeled at the 5′ end with Cy5) was pipetted onto each section, covered by a cover glass, and incubated for 4 h at the optimum hybridization temperature (Table 2). The sections were washed with a wash buffer (hybridization buffer without sodium dodecyl sulfate [SDS]) for 30 min at 52°C. ProLong Gold antifade reagent (Invitrogen, Grand Island, New York) with 4′,6-diamidino-2-phenylindole (DAPI, ∼10 *μ*L) was pipetted onto each section and sealed with transparent nail polish. All FISH probes used in this study are summarized in Table 2, which include the reverse complement of two recently developed polymerase chain reaction (PCR) sense primers to detect *Faecalibacterium* spp. (probe Faecali 698, see “Quantitative real-time PCR” below) and the Phylum Bacteroidetes (probe CFB555f; Muhling et al. 2008), respectively. We also evaluated the abundance of *Faecalibacterium* spp. (using the previously published probe Fprau645), *Lactobacillus* spp. (probe Lac 158), and the *Clostridium coccoides*–*Eubacterium rectale* group (probe Erec 482) (Table 2). Images were obtained from a total of 20 random microscopic fields per time point analyzed (10 microscopic fields were obtained from each section of the fecal blocks) using a Zeiss Stallion digital confocal microscope (Carl Zeiss Microimaging Inc., Thornwood, New York) with a C-apochromat (63× water correction) objective lens. The fecal microorganisms were quantified using an image analysis software in the public domain (ImageJ: http://rsbweb.nih.gov/ij/index.html). One bacterial group (i.e., *Lactobacillus* spp., see below) was either absent or only showed low abundance in most dogs and was therefore counted manually instead. The following formula was used to calculate the number of bacterial cells per gram of wet weight of feces for each microscopic field:

**Table 2:** Oligonucleotides used for FISH and PCR analyses

	Sequence (5′–3′)	Target^1^	Temperature^2^(°C)	References
**FISH probes**				
Lab158	GGTATTAGCATCTGTTTCCA	*Lactobacillus* and *Enterococcus* spp.^3^	50	Harmsen et al. (1999)
Erec482	GCTTCTTAGTCARGTACCG	*Clostridium coccoides*–*Eubacterium rectale* group	50	Franks et al. (1998)
Fprau645	CCTCTGCACTACTCAAGAAAAAC	*Faecalibacterium prausnitzii* and relatives	46	Suau et al. (2001)
Faecali698	GTGCCCAGTAGGCCGCCTTC^4^	*Faecalibacterium* and relatives	50	This study
CFB555f	CCCTTTAAACCCAATDAWTCCGG^5^	Phylum Bacteroidetes	50	Muhling et al. (2008)
**PCR primers**				
FaecaliF	GAAGGCGGCCTACTGGGCAC	*Faecalibacterium* and relatives	60	Garcia-Mazcorro et al. (2012)
FaecaliR	GTGCAGGCGAGTTGCAGCCT^6^	*Faecalibacterium* spp.		

1 As described by listed references (published oligonucleotides) or the Ribosomal Database Project (RDP).

2 Hybridization or annealing temperature.

3 Based on the current RDP, this probe is specific only for *Lactobacillus* spp.

4 This sequence is the reverse complement of the sense PCR primer (FaecaliF) and can detect *in silico* mainly *Faecalibacterium* spp. This oligonucleotide may also detect a proportion of phylogenetically related bacteria such as the genera *Subdoligranulum* (18% of all *Subdoligranulum* in RDP) and *Anaerofilum* (21% of all *Anaerofilum* in RDP). *Anaerofilum* has not been described in intestinal contents.

5 This sequence is the reverse complement of the sense PCR primer to detect the phylum Bacteroidetes developed by Muhling et al. (2008).

6 This sequence can only detect *Faecalibacterium* spp. *in silico* (based on RDP).

Bacterial cells/g feces = bacterial cells in the microscopic field × 33,859 × 600 × 10, where 33,859 is the approximate number of microscopic fields in each paraffin slice of the fecal block (each microscopic field was 14,767 *μ*m^2^ and each paraffin slice is 500 million *μ*m^2^), 600 is the approximate number of paraffin slices (5 *μ*m thick) in each fecal block (∼3 mm deep), and 10 is the factor to multiply for to obtain the numbers of 1 gram of feces (each fecal block was made from 100 mg of feces). In an effort to reduce toxic waste, all hybridizations were performed without formamide in the hybridization buffer. Increasing concentrations of formamide were added to the hybridization buffer only to confirm the obtained counts with different stringency conditions and only in a subset of samples (see below). The probes Faecali 698 and CFB555f were tested for specificity against known strains of *Lactobacillus*, *Streptococcus*, *Enterococcus* (phylum Firmicutes), and *Bifidobacterium* spp. (phylum Actinobacteria).

#### Quantitative real-time PCR

To verify the presence of *Faecalibacterium* in the fecal samples, bacterial DNA was amplified using primers for the 16S rRNA gene of *Faecalibacterium* spp. recently developed in our laboratory (Garcia-Mazcorro et al. 2012; Table 2). The PCR mastermix (10 *μ*L) contained 5 *μ*L of SsoFast EvaGreen supermix (Biorad Laboratories, Hercules, California), 2.2 *μ*L of sterile water, 0.4 *μ*L of each primer (final concentration: 200 nmol), and 2 *μ*L of DNA (5 ng/*μ*L). A melt-curve analysis was performed after all PCR cycles were terminated to confirm the specificity of the primers. The identity (*Faecalibacterium* spp.) of the amplicons was verified using an automated sequence analyzer (ABI PRISM 377 DNA Sequencer, Applied Biosystems, Foster City, California).

### Statistical analysis

The coefficient of variation (%CV) was calculated using the following formula: ([standard deviation/average] × 100). The standard deviation (squared root of variance) was calculated differently to obtain intra- and interindividual %CV. All FISH measurements for each bacterial group were organized in spread sheets with 12 columns (six dogs, two time points) and 20 rows (one for each microscopic field). Intraindividual %CV was calculated using standard deviations from each pair of FISH measurements within a dog (total of 20 pairs, one for each microscopic field). Interindividual %CV was calculated using standard deviations from each set of 12 FISH measurements (six dogs, two time points) within each row (total of 20 sets of 12 FISH measurements). Total %CV was calculated using all 240 FISH measurements (six dogs × two time points × 20 microscopic fields). Similar calculations were employed to obtain intra- and interindividual CV for the relative abundance of pyrosequencing tags (percentage of sequences) using data from the 15 most abundant bacterial families (≥80% of all sequences). The results are reported as medians with interquartile ranges.

## Results

### bTEFAP

**Figure 1:**
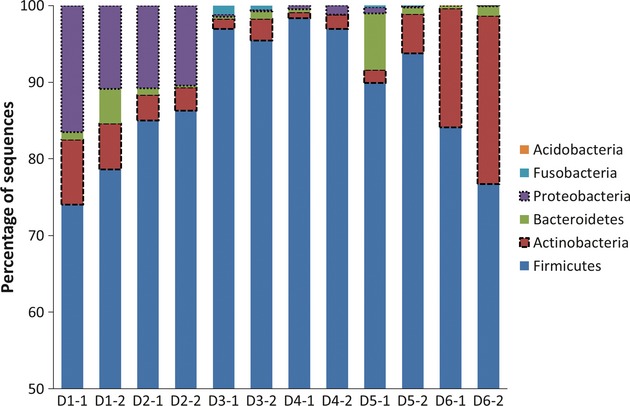
Percentage of bacterial sequences at the phylum level in fecal samples collected from six dogs (D1–D6) and at two time points 15 days apart (-1 and -2). The phyla Actinobacteria and Proteobacteria are highlighted using different border styles for better visualization. Please note that the *y*-axis (percentage of sequences) was modified to also show phyla with low abundance.

The fecal microbiota of all enrolled dogs was dominated by organisms belonging to the phylum Firmicutes, which accounted for ≥75% of all sequences in all dogs (median: 88%, range: 75–98%) (Fig. 1). The second most abundant phylum was Actinobacteria (median: 3%, range: 1–22%), followed by Proteobacteria (median: 1%, range: 0–17%), Bacteroidetes (median: 1%, range: 0–7%), Fusobacteria (only three dogs harbored this phylum at one or both time points with <1% abundance), and Acidobacteria (only three dogs harbored this phylum at one or both time points with <0.1% abundance).

**Figure 2:**
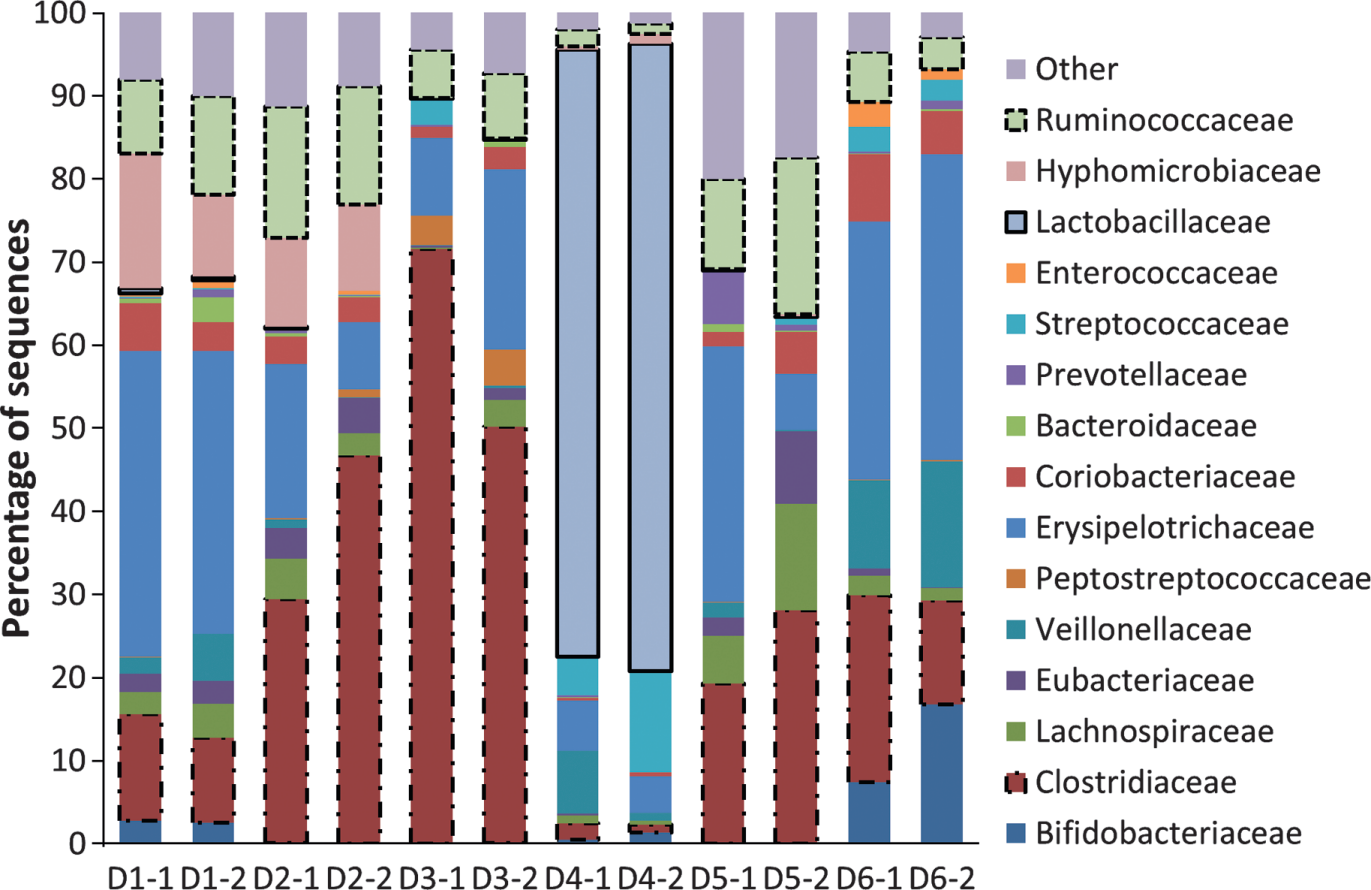
Percentage of bacterial sequences at the family level in fecal samples from six dogs (D1–D6) and at two time points 15 days apart (-1 and -2). The families Clostridiaceae, Lactobacillaceae, and Ruminococcaceae are highlighted using different border styles for better visualization.

Fifteen families comprised ≥80% of the fecal bacterial microbiota in all dogs, and ≥90% of all microbiota in five of the six dogs (Fig. 2). From these 15 families, 10 (67%) families belonged to the phylum Firmicutes, two (13%) families to the phylum Bacteroidetes, two (13%) families to Actinobacteria, and one (7%) family to the phylum Proteobacteria (Fig. 2). The intraindividual %CV (over time) ranged from 2% to 141% (overall median: 55%). In contrast, the interindividual %CV ranged from 62% (family Ruminococcaceae) to 230% (family Lactobacillaceae) (overall median: 145%) (Table S1). Interestingly, the genus *Lactobacillus* was found to be highly abundant in one dog at both time points (Fig. 2, Dog 4, 73% and 75%, respectively, all other dogs had <1%), an observation that was not confirmed using FISH (see “FISH” results below). *Clostridium* was the most abundant genus in all dogs (median: 21%, range: 1–42%) and had an interindividual %CV of 84%. The genera *Faecalibacterium* and its phylogenetically related *Subdoligranulum* showed low abundance in most dogs (only two dogs had 4–7% of *Subdoligranulum*; the rest had ≤1% of either genus) and similarly variable among individual dogs (141 and 143 %CV, respectively). The genus *Anaerofilum*, a bacterial group that is phylogenetically related to both *Faecalibacterium* and *Subdoligranulum*, was detected only at one time point in one dog (<0.1% of all pyrosequencing reads).

### Fish

All FISH results are summarized in Table 3. All dogs had detectable counts of all analyzed bacterial groups except for *Lactobacillus* spp. (probe Lac158), which was either absent (two dogs) or showed low abundance (three dogs harbored <1% of all microbiota at either time point). Only one dog showed relatively high counts of *Lactobacillus* (Dog 2 harbored 5 × 10^8^ counts at one time point, or about 2% of all microbiota, while at the other time point <1% of total counts were observed). These results are not in agreement with the pyrosequencing data (see above), where all dogs had <1% of *Lactobacillus* except for Dog 4, who harbored >70% of *Lactobacillus* at both time points.

**Table 3:** Summary of quantitative results obtained from the FISH analyses^1^

	Abundance of the bacteria (median with interquartile range)^2^			%CV (median with interquartile range)^3^		
		
FISH probe	Number of cells / g wet feces	Log_10_ cells / g wet feces	Percentage of DAPI counts	Intraindividual	Interindividual	Total %CV
Erec482	3.7 (2.2–4.9) × 10^9^	9.6 (9.3–9.7)	22 (13–27)	40 (17–59)	48 (43–54)	48%
CFB555f	4.7 (2.8–9.5) × 10^9^	9.7 (9.5–10.0)	28 (15–41)	38 (18–64)	75 (67–84)	77%
Fprau645	1.0 (4.1 × 10^8^–1.6 × 10^9^) × 10^9^	9.0 (8.8–9.2)	5 (2–7)	28 (11–71)	99 (87–106)	96%
Faecali698	2.8 (1.7–5.1) × 10^9^	9.5 (9.2–9.7)	16 (10–22)	28 (13–56)	68 (65–79)	70%

1 The genus *Lactobacillus* was also quantified using FISH but showed very low abundance (<2%) in all dogs. Therefore, it is not included here.

2 The median and interquartile ranges of the abundance of each bacterial group were calculated from a total of 240 observations (six dogs, two time points, 20 microscopic fields each).

3 The median and interquartile ranges of the coefficients of variation (%CV) were calculated from measurements between the two time points (intraindividual) and among individuals (interindividual). Total %CV was calculated from all 240 observations.

All dogs showed detectable counts of *Faecalibacterium* spp. using both FISH probes with the exception of Dog 3, which did not have any *Faecalibacterium* spp. as estimated using the FISH probe Fprau645. This dog also did not harbor detectable *Faecalibacterium* sequences using quantitative real-time PCR (see below). The use of the probe Fprau645 revealed an overall median of *Faecalibacterium* of 5% of DAPI counts (range: 2–7%), whereas the novel probe Faecali 698 yielded about three times those counts (median: 16% of DAPI counts, range: 10–22%) (Table 3). This difference in proportions between the two FISH probes may be due to populations of *Subdoligranulum* and *Anaerofilum*, although *Anaerofilum* has not been described in intestinal contents (see “Discussion” below). The intra- and interindividual variability was similar for both probes (Table 3).

The numbers and proportions of *Faecalibacterium* spp. (probe Faecali 698) and the phylum Bacteroidetes (probe CFB555f) for subjects harboring the highest amounts of these bacteria were confirmed using increasing concentrations of formamide (results not shown). The specificity of these probes was verified in vitro against strains of *Lactobacillus*, *Bifidobacterium*, *Streptococcus*, and *Enterococcus* spp. All bacteria were fluorescent when using a universal probe (Eub 338) and did not fluoresce when using these two probes.

### Quantitative real-time PCR

Quantitative real-time PCR was used to verify the presence of *Faecalibacterium* in the fecal samples. With the exception of one dog (Dog 3), the use of our *Faecalibacterium*-specific primers yielded the expected 598-bp amplicon in all the samples analyzed. This amplicon corresponds to the positions 698–1291 of the 16S rRNA gene sequence of *Faecalibacterium prausnitzii* (ATCC 27766).

## Discussion

Temporal variations of the intestinal microbiota have been investigated in humans, but limited information is available for other animal species. Also, different microbial identification techniques can yield differing results of the composition of the intestinal microbiota, based on their differences in estimating the abundance of the microorganisms. This study investigated the abundance and short-term temporal variability of fecal microbiota using pyrosequencing and FISH in a population of healthy dogs.

Pyrosequencing (as evaluated using bTEFAP) revealed a distinctive relative abundance of the fecal microbiota in each of the enrolled dogs (Fig. 1), especially at the family level (Fig. 2). As suggested by others (Matsuki et al. 2004; Suchodolski et al. 2008), our results support the possibility that the variation of the intestinal microbiota among individuals (overall median %CV: 145% in this study) is higher than the variation within individuals over time (overall median %CV: 55% in this study). These observations are likely due to the fact that individual dogs had little in common and were exposed to the same environmental conditions (within dogs) during the 15 days period of fecal sample collection. Interestingly, the family with the lowest interindividual variability in abundance (i.e., Ruminococcaceae) has been proposed to be a member of the core human fecal microbiota (Tap et al. 2009), suggesting that some intestinal bacterial groups may also be consistently shared not only among humans but also among other mammals. However, the presence of a core fecal microbiota in nonhuman individuals has not been investigated to date.

The probe Erec482 has previously been used to evaluate the abundance of the *Clostridium coccoides*–*Eubacterium rectale* (Erec) group in several human studies (Marteau et al. 2001; Matsuki et al. 2004; Sokol et al. 2006). Also, one recent study showed that dogs may harbor 9.2–9.6 log_10_ cells/g of wet feces (∼10% of all fecal microbiota, as estimated by DAPI staining) of the Erec group (Jia et al. 2010). Similarly, this study showed an overall median of 3.7 × 10^9^ (9.6 log_10_) cells per g of wet feces of the Erec group (overall median: 22% of all fecal microbiota as estimated using DAPI staining). The difference in the proportions of the Erec group between the study by Jia et al. (10%) and this study (22%) may be due to interindividual differences in total fecal bacterial counts as well as to differences in the study populations. For example, Jia et al. used only Beagle dogs, whereas this study used a more heterogeneous population of dogs. Because the Erec group consists of members of different bacterial groups within the Firmicutes, it is difficult to try to estimate its true proportion based on pyrosequencing results. For example, an *in silico* analysis of specificity using the Probe_Match tool of RDP (all analyses using probe match were performed with 0 errors allowed) shows that the Erec 482 probe can only detect the genus *Blautia* (family Incertae sedis XIV) and several genera within the family Lachnospiraceae (e.g., *Butyrivibrio, Coprococcus, Roseburia, Dorea, Anaerostipes*). Together, all these genera comprise ∼4% of all pyrosequencing tags in this study. This matter is further complicated because of recent reclassifications of some of these bacterial groups (e.g., Liu et al. 2008).

Recently, the phylum Bacteroidetes has received considerable interest due to its potential involvement in the pathogenesis of obesity (Ley et al. 2005; Turnbaugh et al. 2006). One recent study showed that this phylum may comprise a median of 9.3% (Probe Bac303, range: 5.6–13.9%) or 5.6% (Probe CFB286, range: 3.3–32.0) of all fecal microbiota in healthy humans (Hoyles and McCartney 2009), as estimated using FISH. These probes were developed by Manz et al. (1996) and Weller et al. (2000), respectively. One recent study suggested that Bacteroidetes shows a low abundance (<1% of all microbiota) in the feces of dogs (Jia et al. 2010) using another FISH probe (CFB719) also developed by Weller et al. (2000). Using the reverse complement of a sense PCR primer developed by Muhling et al. (2008), this study suggests that Bacteroidetes may be more abundant in feces of dogs (median: 28% of DAPI counts). These results are in concordance with another study showing that the order Bacteroidales is abundant (∼30% of all sequences) in intestinal contents of the colon of dogs (Suchodolski et al. 2008), using 16S rRNA gene clone libraries. Also, one recent study used meta-genomics and showed that this phylum comprises ∼35% of all fecal microbiota in dogs (Swanson et al. 2011). However, pyrosequencing results in this and other studies (Handl et al. 2011) suggest that Bacteroidetes is low abundant (∼2% of all sequences) in feces of dogs. Published results in humans with respect to the proportion of the Bacteroidetes also vary widely (Armougom and Raoult 2008). Studies are needed to determine the identities and exact proportions of members from the Bacteroidetes in feces of dogs.

The bacterium *F. prausnitzii* has received increased attention because of its potential protective role against inflammatory bowel disease in humans (Sokol et al. 2008). Interestingly, one study suggested that the *Faecalibacterium* species found in dogs may not be *F. prausnitzii* (Suchodolski et al. 2008), based on the phylogenetic affiliation of near-full-length 16S rRNA gene sequences belonging to a canine clone (C2-02) and a human strain (AJ270469). We have designed two PCR oligonucleotides that are capable of amplifying the genus *Faecalibacterium* (as evaluated by *in silico* analysis and sequencing of amplicons, Table 2). From these two primers, we chose the sense primer (FaecaliF) as candidate for a FISH probe because it can *in silico* detect 22% more *Faecalibacterium* sequences than the anti-sense primer (81% vs. 59% of all *Faecalibacterium* sequences in RDP). However, this sense primer may also detect some phylogenetically related bacteria, the genus *Subdoligranulum* (Holmstrom et al. 2004) and the poorly described genus *Anaerofilum* (Zellner et al. 1996). Importantly, the presence of *Anaerofilum* in intestinal contents has not been described in the literature. To our knowledge, the only other FISH probe available to detect intestinal *Faecalibacterium* is the probe Fprau645 that was developed by Suau et al. (2001). This probe does not detect any *Subdoligranulum* or *Anaerofilum* (based on RDP) and can detect in silico many *Faecalibacterium* (78% of all *Faecalibacterium* sequences in RDP). Using this probe, Suau et al. (2001) suggested that *F. prausnitzii* and its relatives may comprise ∼17% of all fecal microbiota (as estimated by DAPI staining) of healthy humans. Using the same FISH probe, another, more recent study, also showed that *Faecalibacterium* and relatives comprise 14–17% of all fecal microbiota in humans (Benus et al. 2010). In contrast, in this study, the use of this FISH probe revealed that *Faecalibacterium* and relatives comprise ∼5% of all fecal microbiota in the enrolled dogs, while our FISH probe revealed about three times this proportion (∼16% of all microbiota). This difference in proportions between the two FISH probes may be due to populations of *Subdoligranulum*. Interestingly, pyrosequencing revealed that both *Faecalibacterium* and *Subdoligranulum* were present in low abundance in most dogs (only two dogs had 4–7% of *Subdoligranulum*, the rest of the dogs had ≤1% of either genus), whereas the genus *Anaerofilum* was detected only in one dog and at one time point (<0.1% of all pyrosequencing reads). As with other bacterial groups, studies are needed to determine the exact abundance of *Faecalibacterium* and its relatives to help determine their role during intestinal disease in dogs and other animal species.

This study shows some discrepancies between the pyrosequencing and the FISH results, especially with regard to the abundance of the bacterial groups. This was expected, in part, because pyrosequencing relies on PCR to amplify genomic targets and may therefore underestimate the true abundance of all members of the microbiota. Also, the primers used in this study for pyrosequencing can amplify the variable regions V1 to V3 of the 16S rRNA gene, whereas some of our FISH probes (e.g., Faecali 698) target regions outside of these regions. However, 454-pyrosequencing and other high-throughput techniques can also provide the relative proportions of the different bacterial groups, which ultimately provide useful information with regard to a change in the relative abundance of the microorganisms.

This study used two oligonucleotides to determine the abundance of the phylum Bacteroidetes (probe CFB555f) and *Faecalibacterium* relatives (probe Faecali 698) in feces of dogs using FISH. However, the ability of these two probes to hybridize with related and unrelated bacteria has been only evaluated *in silico* either by our group (Faecali 698, this study) and by Muhling et al. (2008) (probe CFB555f). Muhling et al. went a step further and created a clone library using a primer pair containing this oligonucleotide, and showed that all sequences produced in the clone libraries were derived from members of the Bacteroidetes. In this study, we additionally showed that these two probes do not hybridize with members of two different phyla *in vitro*, the Firmicutes (*Lactobacillus*, *Streptococcus*, and *Enterococcus* spp.) and the Actinobacteria (*Bifidobacterium* spp.). Further studies are nevertheless warranted to investigate more extensively the specificity *in vitro* of these two oligonucleotides using FISH.

In summary, normal temporal variations of the intestinal microbiota have been studied mainly in humans, with little information available in other animal species. Pyrosequencing and FISH are different molecular techniques that are commonly used in gut microbial ecology to characterize the intestinal microbiota and its changes in response to external influences. Our results suggest that these two techniques yield different abundances of some of the most predominant fecal microbiota in healthy dogs, including *Faecalibacterium*, a bacterial group that has consistently been shown to be depleted during intestinal disease, and Bacteroidetes, a phylum which has been suggested to play an important role in energy harvesting and obesity. These abundances seem to vary more among dogs than within dogs within a period of 2 weeks, although this seems also to be dependent on the technique employed and the specific bacterial group analyzed. These observations should be considered when evaluating the effect of agents on the canine intestinal microbiota.

## Supporting Information

**Table S1.** Relative abundance (proportions of pyrosequencing reads) of the most predominant bacterial families in fecal samples from six dogs (D1–D6) and at two time points 15 days apart (-1 and -2) with interindividual coefficients of variation (%CV).
