# Supplementary material for: Abundance and short-term temporal variability of fecal microbiota in healthy dogs
Source: Microbiologyopen. 2012 Sep 3;1(3):340–7. doi: 10.1002/mbo3.36 (PMC3496977; doi:10.1002/mbo3.36)
Supplement: Supplementary file 1 [file mbo30001-0340-SD1.doc]

| Bacterial Family | D1-1 | D1-2 | D2-1 | D2-2 | D3-1 | D3-2 | D4-1 | D4-2 | D5-1 | D5-2 | D6-1 | D6-2 | **%CV** |
| --- | --- | --- | --- | --- | --- | --- | --- | --- | --- | --- | --- | --- | --- |
| Ruminococcaceae | 8.9 | 11.9 | 15.8 | 14.3 | 5.8 | 7.8 | 2.0 | 1.3 | 10.8 | 18.9 | 6.1 | 3.9 | **62** |
| Erysipelotrichaceae | 36.8 | 34.1 | 18.6 | 8.1 | 9.4 | 21.7 | 6.1 | 4.4 | 30.8 | 6.8 | 31.0 | 36.8 | **64** |
| Coriobacteriaceae | 5.7 | 3.4 | 3.3 | 3.0 | 1.3 | 2.7 | 0.3 | 0.5 | 1.7 | 5.0 | 8.1 | 5.2 | **69** |
| All other families | 8.1 | 10.0 | 11.3 | 8.8 | 4.4 | 7.3 | 2.0 | 1.3 | 20.0 | 17.4 | 4.7 | 2.9 | **72** |
| Clostridiaceae | 12.8 | 10.2 | 29.4 | 46.7 | 71.6 | 50.1 | 2.0 | 0.9 | 19.3 | 28.1 | 22.6 | 12.5 | **83** |
| Lachnospiraceae | 2.7 | 4.1 | 4.8 | 2.7 | 0.2 | 3.2 | 0.9 | 0.4 | 5.8 | 12.9 | 2.3 | 1.5 | **99** |
| Eubacteriaceae | 2.2 | 2.7 | 3.7 | 4.2 | 0.2 | 1.4 | 0.3 | 0.1 | 2.2 | 8.7 | 0.8 | 0.1 | **112** |
| Veillonellaceae | 1.9 | 5.6 | 1.1 | 0.1 | 0.1 | 0.4 | 7.5 | 0.9 | 1.9 | 0.1 | 10.6 | 15.2 | **131** |
| Hyphomicrobiaceae | 16.2 | 9.8 | 10.7 | 10.4 | 0.0 | 0.0 | 0.5 | 1.2 | 0.0 | 0.2 | 0.0 | 0.0 | **145** |
| Streptococcaceae | 0.2 | 0.2 | 0.1 | 0.0 | 3.2 | 0.0 | 4.6 | 12.0 | 0.0 | 1.0 | 3.0 | 2.5 | **156** |
| Bacteroidaceae | 0.6 | 3.0 | 0.4 | 0.1 | 0.0 | 0.7 | 0.1 | 0.0 | 0.9 | 0.2 | 0.1 | 0.3 | **157** |
| Enterococcaceae | 0.4 | 0.9 | 0.1 | 0.5 | 0.0 | 0.0 | 0.1 | 0.1 | 0.1 | 0.0 | 2.9 | 1.3 | **163** |
| Bifidobacteriaceae | 2.8 | 2.5 | 0.1 | 0.0 | 0.0 | 0.1 | 0.5 | 1.4 | 0.0 | 0.0 | 7.4 | 16.8 | **188** |
| Peptostreptococcaceae | 0.1 | 0.0 | 0.1 | 1.0 | 3.5 | 4.3 | 0.0 | 0.0 | 0.1 | 0.0 | 0.1 | 0.2 | **189** |
| Prevotellaceae | 0.1 | 0.9 | 0.4 | 0.1 | 0.2 | 0.1 | 0.2 | 0.0 | 6.4 | 0.7 | 0.3 | 1.0 | **204** |
| Lactobacillaceae | 0.6 | 0.5 | 0.2 | 0.0 | 0.1 | 0.2 | 73.0 | 75.4 | 0.1 | 0.1 | 0.0 | 0.0 | **230** |

**Supplementary Table 1** Relative abundance (proportions of pyrosequencing reads) of the most predominant bacterial families in fecal samples from 6 dogs (D1 to D6) and at two time points 15 days apart (-1 and -2) with inter-individual coefficients of variation (%CV).
